# A Self-Balancing Nanovolt Potentiometric System for Thermometry and Calorimetry

**DOI:** 10.6028/jres.080A.067

**Published:** 1976-08-01

**Authors:** Shu-Sing Chang

**Affiliations:** Institute for Materials Research, National Bureau of Standards, Washington, D.C. 20234

**Keywords:** Automation, automatic potentiometry, calorimetry, Diesselhorst ring, potentiometer, programmable potentiometer, self-balancing potentiometers, thermometry

## Abstract

The principle of a self-balancing potentiometric system is described. The principle is applied to the modification of an existing manually operated thermo-free, low voltage potentiometer consisting of Diesselhorst ring elements. The modification involves the addition of reed relays which enable the potentiometer voltage to be set by digital signals. By incorporating a digital voltmeter, or an analog-to-digital converter, and a nanovolt amplifier with the modified potentiometer, self-balancing of the potentiometer may be achieved through either hardware logic implementation or direct digital control from a minicomputer. The resolution of this self-balancing potentiometric system for a full scale input of 100 mV is about one to 10 parts in 10^8^. With real-time digital processing of the data, resolution of about 1 nV or better has been achieved for slowly changing input signals. The overall accuracy of the system is better than 10 ppm for voltage measurements and about 1 ppm for voltage ratio or resistance measurements.

## 1. Introduction

In high precision low temperature calorimetry covering the temperature range from 4 to 400 K, a platinum resistance thermometer is often used as the temperature indicating instrument. The resistance of the thermometer may be measured either by a bridge or by a potentiometer. Automated high resolution dc and ac bridges have been developed and used in thermometry and calorimetry [1–4]^1^. In the potentiometric method the resistance of a four terminal resistor is obtained from the ratio of the potential drop developed across the unknown resistance to that across a series-connected standard resistor of known value. The advantage of the potentiometric method over the bridge method is that with the former the measurement is independent of the lead wire resistance. This is especially important at low temperatures where the lead wire resistance may be a couple of orders of magnitude greater than the resistance of the thermometer to be measured. An added advantage in calorimetry is that the potentiometer may be used also to measure the energy supplied to the calorimeter. The present work describes the principle and an example of an automated potentiometric system with high resolution and high sensitivity suitable for platinum resistance thermometry and calorimetry work, by the modification of an existing manually operated thermo-free potentiometer into a programmable potentiometer.

## 2. Principle

**Figure 1:**
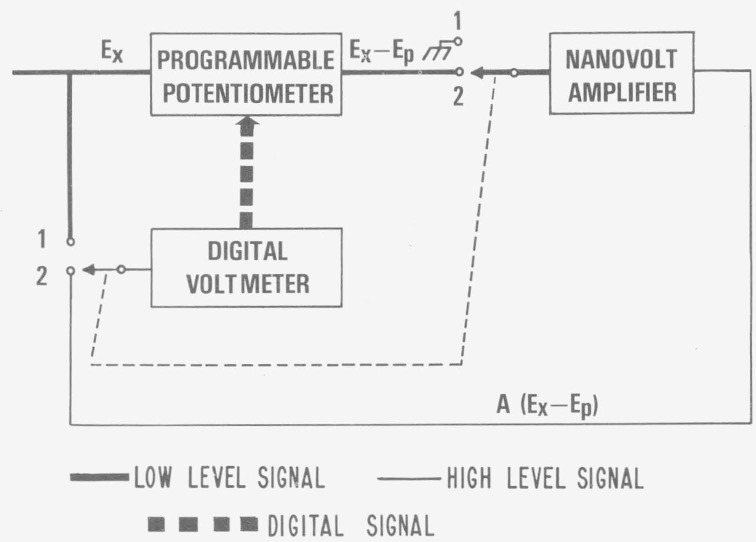
Principle of automated potentiometric system.

The principle of this present approach to a high resolution automatic potentiometric measurement is outlined in figure 1 and is described by the following sequence of operations.

1. With the ganged switch at position 1, the galvanometer or detector output of the programmable potentiometer, POT, is open and the input to the nanovolt amplifier, NVA, is shorted.
2. A digital voltmeter, DVM, or an analog-to-digital converter is used to sample the unknown voltage, *E*_x_. The first few most significant figures of the DVM are then transferred digitally to the POT to generate a voltage *E_p_.*
3. The ganged switch changes to position 2. This connects the NVA input to the output of the POT. The NVA amplifies the POT imbalance signal, *E_x_*−*E_p_* by an amplification factor *A.* The DVM input is now connected to read the output of the NVA, *E_n_=A*(*E_x_*−*E_p_*).

The combination of the two DVM readings, *E_p_* and *E_n_*, yields a potential readng of *E_x_=E_p_+E_n_/A* to a high degree of resolution. In the following example an automated potentiometric system with a resolution of the order of one to 10 parts in 10^8^ and a sensitivity of about 1 nV is demonstrated.

## 3. Programmable Potentiometer With Diesselhorst Rings

One commercially available manual potentiometer is easily adaptable to the principle outlined above. It is a double six-dial potentiometer^2^ of thermo-free design [5]. The four most significant decades are composed of Diesselhorst rings [6]. These four decades are divided into two sections, each with its own power supply. The least significant two decades are composed of Lindeck elements. The maximum potentiometer voltage is 0.1 V.

**Figure 2:**
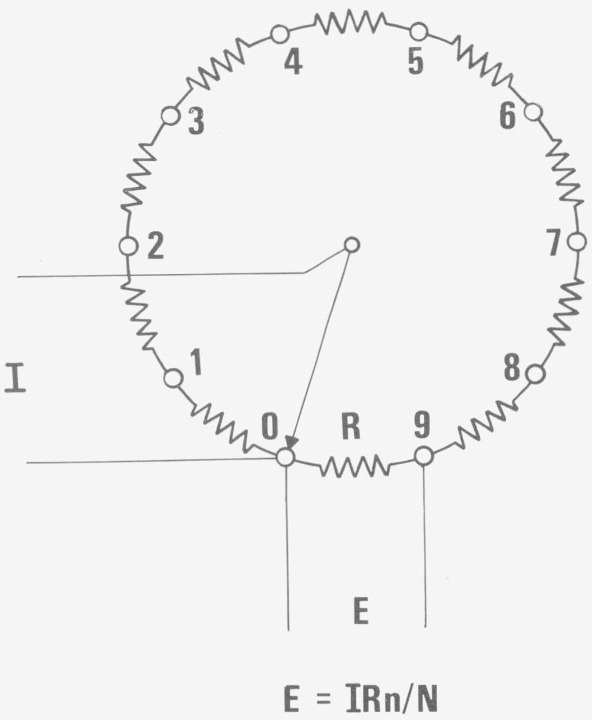
Diesselhorst ring.

**Figure 3:**
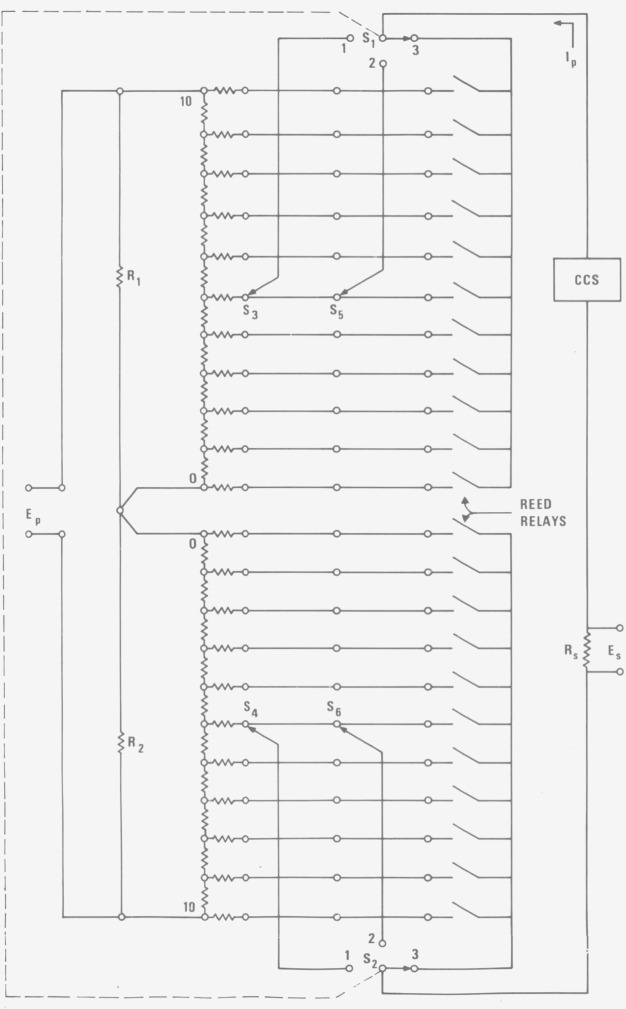
Schematic diagram of a potentiometer with two Diesselhorst rings.

A Diesselhorst ring consists of *N* resistors, each of resistance *R* connected in series. In figure 2, a ring of 10 resistors is shown. The junctions between the resistors are labeled from *n*=0 to *N*−1 starting from the fixed current lead. The ring current, *I* flows between position 0 and a position *n* on the ring, selectable by a movable pointer. The potential drop *E* developed across the resistor between positions 0 and *N*−1 is then given as *IR n/N*,

In general one decade of potential values is generated by one such ring. Several rings of successive decades may be connected in series to form a potentiometer. A schematic diagram of one of the sections of the potentiometer with modifications is shown in figure 3. The values of the ring elements *R*_1_ and *R*_2_ are related such that *R*_1_=10 *R*_2_ where *R*_1_ is in the most significant decade.

The potential, *E_p_*, of the potentiometer is connected in series with but opposing the unknown voltage, *E_x_*, to be measured. A galvanometer or a nanovolt amplifier may be connected in series with *E_x_* and *E_p_* as a null detector or as an unbalanced signal indicator. There are no sliding contacts involved in this (*E_x_*−*E_p_*)-detector-potentiometric circuit. Any change in the switch resistance and thermal emf is restricted mainly to the potentiometer current, *I_p_*, circuit.

If a highly regulated constant current supply, CCS, is used to supply *I_p_* it minimizes the effects from the irreproducibility of the switch resistance and from the thermal emf generated in the switches. Therefore, the quality of the switches to be used in conjunction with a CCS is not critical, and ordinary reed relays in dual-in-line packages may be used in place of the massive rotary switches in the original potentiometer.

A set of reed relays is connected in parallel with the decade positions of each pair of the original rotary switches *S*_3_–*S*_5_ and *S*_4_–*S*_6_. Simple logic circuits permit the activation of only one relay in each decade. These sets of relays provide a programmable setting of the potential *E_p_* at position 3 of the ganged selector switch *S*_1_–*S*_2_, in addition to the original manual settings of *E_p_* at positions 1 and 2.

**Figure 4:**
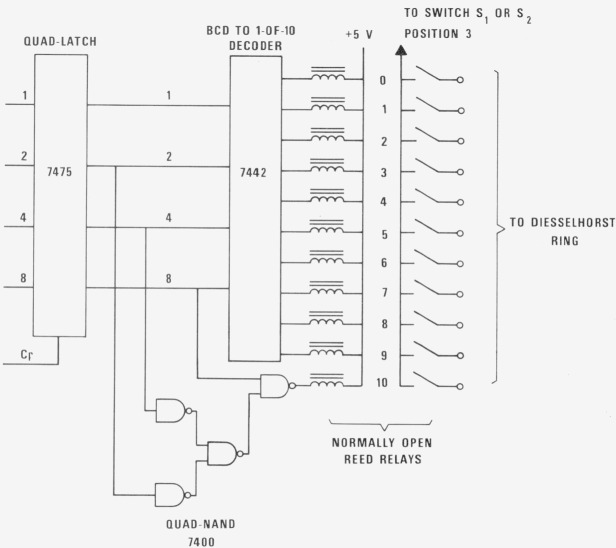
Schematic diagram of programmable control of Diesselhorst ring.

The schematic diagram of the logic circuit to control one of the Diesselhorst rings is shown in figure 4. Identical control circuits are used for other decades. The clock pulse, *C_p_*, is activated when a change of potentiometer setting, *E_p_* is desired. During the clock pulse, the incoming binary coded decimal, BCD, signal passes through the latch to the decoder which activates one of the ten relays (0 to 9). The potentiometer setting will remain latched at the value when the clock pulse is deactivated.

Position 10 and its associated circuit is not required for ordinary potentiometric operation. However, the presence of the 10th position and the ability to reverse the polarities of the individual power supplies of the potentiometer make the auto-calibration or dial-to-dial comparison of the potentiometer easier.

The relays at position 10, activated by any signal designated from 10 to 15, also avoids the open condition to the CCS, even in case the decoder receives a random or noisy signal other than for 0 to 9. To further prevent the CCS from experiencing an open circuit, normally closed relays are placed between positions 3 and 1 or 3 and 2 of ganged switch *S*_1_*–S*_2_. These relays are activated directly by the 5 V power supply of the potentiometer relay controller. Therefore the CCS will not seen an open circuit even if the power to the controller is off.

The resistors between the ring elements and the decade switches (see fig. 3) are used to provide a constant resistive load to a potentiometer power supply (usually a battery or a constant voltage supply) and thus provide a constant *I_p_* at different *E_p_* settings. However, inexactness of the adjustment in the values of these resistors may require read-justment of *I_p_* for every change of *E_p_.* With the CCS, these resistors become unimportant and may be eliminated entirely. The use of CCS also eliminates the need for frequent *I_p_* standardization when batteries or constant voltage supplies are used to supply the *I_p_*. The potentiometer current, *I_p_* may be monitored by the voltage drop, *E_s,_* developed across the current sensing resistor, *R_s_.*

## 4. Constant Current Supply

Highly stable constant currents required by the potentiometer may be supplied by either commercially available CCS’s with photogalvanometer feedback control or CCS’s built around a highly stable, low noise operational amplifier.

**Figure 5:**
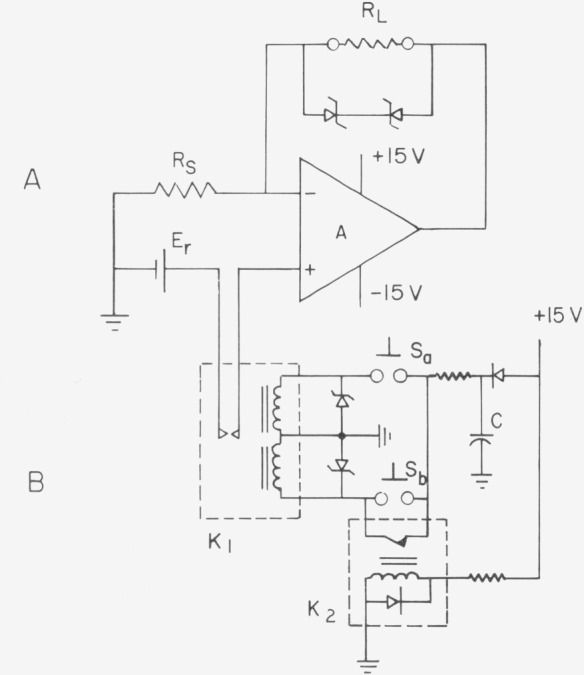
Schematic diagram of constant current supply.

The schematic of a simple constant current supply is shown in figure 5A. By properly choosing the components, a long term stability of better than 1 ppm h^−1^ and 10 ppm per month may be achieved while the CCS is operating in ambient temperature. The operational amplifier, *A*, should have low input noise (less than 1 *µ*V), low input offset drift (less than 5 *µV* per month), low bias current (less than 1 nA), and high input impedance (greater than 10^10^ Ω). The reference voltage, *E_r_*, may come from an unsaturated standard cell at ambient temperature, a saturate standard cell in a thermostat, or other stable voltage sources. The current sensing resistor or resistive network, *R_s_* should have a low temperature coefficient and a low thermal emf against copper. The value of *R_s_* is selected or adjusted to yield the desired current *I=E_r_/R_s_.* A capacitor in the order of 0.005 to 0.5 *µ*F connected in parallel with *R_s_* may sometimes reduce the noise appearing at the output. The connections between *A*, *E_r_* and *R_s_* are made with copper binding posts or terminal strips with copper shorting links and with low thermal solder to reduce the generation of thermal emf. The zener diodes connected across the output load, *R_L_*, prevent a large current drain from *E_r_* in case the load is open.

In a different configuration than shown in figure 5A, the resistive load *R_L_* may be placed between the ground and the common point of *E_r_* and *R_s_* while the output of the amplifier is connected directly to the negative input. However, this configuration works well only if the voltage developed across the load is much less than 1 V, otherwise the performance of the amplifier deteriorates because of the high common mode voltage applied to both inputs.

In order to protect the standard cell *E_r_* from excessive discharge in case of electrical power failure, a low thermal emf (<1 *_µ_*V) bistable magnetic latching relay is installed in series in the standard cell circuit. The relay control circuit is shown in figure 5B. When the ac power to the ± 15 V power supply is turned off, the normally closed reed relay *K*_2_ causes the energy stored in capacitor *C* to be discharged through the “off” coil of the latching relay, *K*_1_, and thus disconnects the standard cell. The standard cell connection may be restored manually by the momentary switch *S_a_*, when the ac power is on. Switch *S_b_* may be used to disconnect the standard cell manually. The diodes or zener diodes across the relay coils are used to protect the relays, especially the latching relay.

## 5. Low Level Signal Multiplexers

The relays used in the system to select different inputs to the DVM generate less than 0.5 *µ*V of offset thermal emf, and that used for the input to the nanovolt amplifier less than 50 nV. Under normal operational conditions these offset voltages are relatively stable. Both types of relays are of bistable magnetic latching construction. A pulse duration of 1 ms is sufficient to change the state of the relay. The very low duty cycle with such a short pulse keeps the energy dissipation in the relay coil to a minimum and produces negligible temperature differential between the relay contacts. These relays have copper leads which further reduce the thermal emf at the relay connections. Although the two types of relays used in this system and several other types of latching-type relays tested have satisfactory thermo-electrical characteristics, their mechanical reliability seems to be low. The biasing magnets in the relays are susceptible to damages due to overdrive. This may cause failures of the relays to latch onto one of the states without constant coil current. A low thermal emf silver-silver alloy rotary switch used in the manually operated potentiometric system is retained as the selector for low level signals (*E_x_*) to the potentiometer. A rotary solenoid has been added to this rotary switch so that its position may be selected by digital signals.

When the potentiometer is used for resistance measurements, such as in the case of resistance thermometry, the stray and thermal emf in the potentiometer-*E_x_*-detector circuit may be obtained by reversing the polarities of all the CCS’s used for the potentiometer and thermometer. This may be accomplished with DPDT relays with common open periods. The connections associated directly with the *E_x_* measurement path, such as the potentiometer input selector and the nanovolt amplifier input relay, should remain undisturbed during the reversal measurement.

## 6. Program for Self-Balancing Potentiometric Measurements

Hardware logic circuits have been constructed and tested to operate the potentiometric system according to the procedures outlined in the Principle section. Only a few momentary switches or a sequential switch are required to carry out the procedure. However, as a minicomputer became available, a simpler digital interface to the central processing unit has been built. This provides far more versatile operation of the system through software implementations.

**Figure 6:**
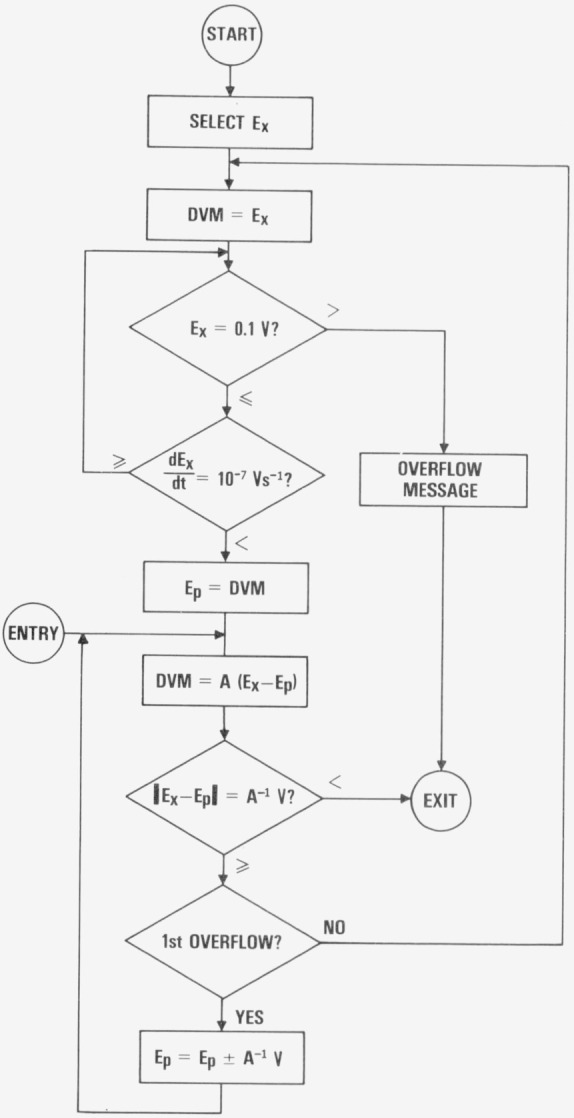
Flow chart of self-balancing potentiometer program.

A typical software program for self-balancing procedure is illustrated by the flow chart, figure 6. The values of the controlling parameters in the diagram may be changed to suit the application. The program begins with the low level signal scanner selecting an unknown voltage source *E_x_*, to be measured. *E_x_* is first examined by the DVM to a resolution of 1 *_μ_*V. If *E_x_* is greater than 0.1 V, the maximum voltage of *E_p_* the potentiometric measuring procedure is bypassed. If *E_x_* is less than 0.1 V and the rate of change of *E_x_* is less than 10^−7^ V s^−1^, potentiometer setting, *E_p_* is set to the nearest 10 *_μ_*V of the value *E_x_* as indicated by the DVM. The DVM is then shifted to read the output of the NVA, which amplifies the unbalanced signal, *E_x_−E_p_* of up to ±10 *_μ_*V by an amplification factor *A.* The full scale reading of the NVA is usually equal to the last digit of the programmable POT. When the NVA reading is within its range, the automated voltage measurement on *E_x_* is completed. The observed *E_x_* value is the sum of the rounded first DVM reading or the POT setting, *E_p_* and the second DVM reading divided by the amplification factor of the NVA, thus *E_x_=E_p_+*[*A*(*E_x_−E_p_*)]/*A.*

When overflow of the NVA occurs, *E_p_* may be increased or decreased by one last digit or 10 *_μ_*V depending upon the direction of the overflow. If an overflow condition persists, it indicates that the rate of change of *E_x_* is greater than the speed of the potentiometric measurement. The POT is bypassed and reset when the DVM indicates that *dE_x_/dt* is small enough. On the other hand, if *E_x_* remains rather steady, further potentiometric measurements may be initiated at the additional entry point without disturbing the *E_p_* setting and the low level switches.

## 7. Characteristics and Applications

The accuracy and the stability of the entire potentiometric system depends upon many factors, such as the stability of the CCS’s, the temperature coefficient of the ring resistors in the Diesselhorst ring, the rate of ambient temperature fluctuations, and to a lesser degree upon the gain stability of NVA and of DVM, etc.

The sensitivity of the system is limited by the noise of the NVA to about 5 nV. However, for slowly changing signals, it is possible to make average NVA readings every 30 s. A moving collection of odd-numbered equally spaced readings, for 5 to 10 min is then subjected to a simple least-squares evaluation of a quadratic equation *E*(*t*)*=E*_0_*+at+bt*^2^. *E*_0_ is the estimated signal at the midpoint of the least-square period (*t*=0). The first derivative ‘*a*’ in the quadratic equation is identical to the least-square evaluated derivative ‘*a*’ in the linear equation *E*(*t*) *=E*′_0_+*at.* The moving average method acts as a filter with a long time constant. Thus the short term noise of the NVA may be filtered out, and the system is capable of resolving signals to better than 1 nV from a signal of less than 100 mV. However, long term fluctuations, such as those caused by the temperature controlled oven for the standard cells and current sensing resistors in the CCS, may still be detected. For steady or monotonically changing signals, the successive change of the signs of the second derivative ‘*b’* of the quadratic equation indicates the approach of the limit of measurement capability of the potentiometric system.

The accuracy of the voltage measurement by this system is about 10 ppm, even after the application of dial-to-dial autocalibration factors and the calibration of the main resistor *R*_1_ because the potentiometer current sensing resistor *R_s_* in the potentiometer is adjustable in 10 ppm steps. However, for ratio or relative measurements, the accuracy is better than 1 ppm.

This potentiometric system has been used principally to measure temperatures in automated precision adiabatic calorimetry. Details of the automated calorimetric procedure and the results obtained with the automated system on the heat capacity data from 5 to 380 K, for *α*-Al_2_O_3_, poly-(vinyl chloride) and poly(chlorotrifluoroethylene) are reported elsewhere [7]. The sample of *α*-Al_2_O_3_ used is a National Bureau of Standards, Standard Reference Material (SRM) 720, Synthetic Sapphire, for calorimetry. Its enthalpy and heat capacity values have been previously certified from 273 to 2250 K.

The temperature of the calorimeter is indicated by the resistance of a platinum resistance thermometer whose resistance is about 35 Ω at 373 K and 0.02 Ω at 4 K. A thermometer current of 1 to 2 mA is generally used, except at liquid helium temperatures where a current of up to 10 mA may be used to increase the sensitivity. These currents yield a thermometric signal from 200 *_μ_*V to 80 mV. The thermometer current is also supplied by a CCS with photogalvanometer feedback control or a highly stable, low drift operational amplifier and is monitored by the potential drop across a 10 Ω standard resistor. With the above-mentioned procedure to achieve a resolution of about 1 nV for the potentiometric system, temperatures may be resolved to 10^−5^ K at temperatures above 50 K. The overall stability of the entire calorimetric temperature measurement may be judged from the stability of the thermometer current. The current seems to change slowly within 1 ppm h^−1^ and cycles within 10 ppm in 24 h. This cyclical behavior is probably due to the relatively high temperature coefficient of the ring resistors and the current sensing resistor, *R_s_* which follow a day to night ambient temperature change of 1 to 2 K.

The energy input to the calorimeter is also measured by the automated potentiometric procedure, however, the requirements in the energy measurement are less demanding than those of the temperature measurement.

## 8. Conclusion

It was found that the principle outlined in the introduction is a practical and workable one. The resolution of the potentiometric system has actually been increased by the automation and the digital data treatment outlined. In many cases of automation, there have often been trade-offs between precision and convenience.

The NVA used in the present system is stable to about 0.1 percent. By using a unit with 10 ppm stability (now available), it should be possible to increase the range and resolution of the amplifier by means of incorporating two decades of programmable Diesselhorst rings. These rings should be made of resistors with a low temperature coefficient. A microprocessor may be incorporated together with the programmable Diesselhorst rings, CCS, and NVA as one integral instrument to yield up to an eight-digit display of the unknown potential without the use of an external computer.

The author thanks R. J. Carpenter for assistance in the design, construction, and testing of various logic and interface circuits and for many helpful discussions, F. I. Mopsik and P. T. Olsen for discussions on the applications of operational amplifiers and C. H. Pearson for assistance in the assembly of the system.

## Footnotes

1 Figures in brackets indicate the literature references at the end of this paper

2 Commercial materials are identified in this paper to adequately specify the experimental procedure. Such identification does not imply recommendation or endorsement by the National Bureau of Standards.
